# Identification, characterisation and expression analysis of natural killer receptor genes in *Chlamydia pecorum* infected koalas (*Phascolarctos cinereus*)

**DOI:** 10.1186/s12864-015-2035-x

**Published:** 2015-10-15

**Authors:** Katrina M. Morris, Marina Mathew, Courtney Waugh, Beata Ujvari, Peter Timms, Adam Polkinghorne, Katherine Belov

**Affiliations:** Faculty of Veterinary Science, University of Sydney, Camperdown, NSW Australia; Institute of Health and Biomedical Innovation, Queensland University of Technology, Kelvin Grove, Brisbane, QLD Australia; Faculty of Science, Health, Education and Engineering, University of the Sunshine Coast, Sippy Downs, QLD Australia; Centre for Integrative Ecology, School of Life and Environmental Sciences, Deakin University, Waurn Ponds, Geelong, VIC Australia

**Keywords:** Koala, *Phascolarctos cinereus*, Chlamydia, Natural killer cells

## Abstract

**Background:**

Koalas (*Phascolarctos cinereus*), an iconic Australian marsupial, are being heavily impacted by the spread of *Chlamydia pecorum*, an obligate intracellular bacterial pathogen. Koalas vary in their response to this pathogen, with some showing no symptoms, while others suffer severe symptoms leading to infertility, blindness or death. Little is known about the pathology of this disease and the immune response against it in this host. Studies have demonstrated that natural killer (NK) cells, key components of the innate immune system, are involved in the immune response to chlamydial infections in humans. These cells can directly lyse cells infected by intracellular pathogens and their ability to recognise these infected cells is mediated through NK receptors on their surface. These are encoded in two regions of the genome, the leukocyte receptor complex (LRC) and the natural killer complex (NKC). These two families evolve rapidly and different repertoires of genes, which have evolved by gene duplication, are seen in different species.

**Methods:**

In this study we aimed to characterise genes belonging to the NK receptor clusters in the koala by searching available koala transcriptomes using a combination of search methods. We developed a qPCR assay to quantify relative expression of four genes, two encoded within the NK receptor cluster (*CLEC1B, CLEC4E*) and two known to play a role in NK response to Chalmydia in humans (*NCR3, PRF1*).

**Results:**

We found that the NK receptor repertoire of the koala closely resembles that of the Tasmanian devil, with minimal genes in the NKC, but with lineage specific expansions in the LRC. Additional genes important for NK cell activity, *NCR3* and *PRF1*, were also identified and characterised. In a preliminary study to investigate whether these genes are involved in the koala immune response to infection by its chlamydial pathogen, *C. pecorum*, we investigated the expression of four genes in koalas with active chlamydia infection, those with past infection and those without infection using qPCR. This analysis revealed that one of these four, *CLEC4E,* may be upregulated in response to chlamydia infection.

**Conclusion:**

We have characterised genes of the NKC and LRC in koalas and have discovered evidence that one of these genes may be upregulated in koalas with chlamydia, suggesting that these receptors may play a role in the immune response of koalas to chlamydia infection.

## Background

Koalas (*Phascolarctos cinereus*) are an iconic Australian species and the last surviving of the family *Phascolarctidae*. Although they are listed as a species of least concern [1], the koala population is in decline across the majority of its range, and the species is listed as vulnerable in some states by the Australian government [2]. One of the greatest threats to koalas is the spread of infectious diseases. Modelling has demonstrated that control of disease is the most important factor for stabilising peri-urban koala populations [3]. The most significant disease affecting koala populations is *Chlamydia pecorum*, which has prevalence as high as 72–100 % in some mainland koala populations [2]. This sexually transmitted disease has had devastating effects on some populations of koalas. Infection of the mucosa of the eye causes inflammation and can lead to blindness while infection of the urogenital tract can cause cystitis, ulceration, infertility and death [2]. However response to this disease varies greatly; while many koalas with high infectious loads display no symptoms, others with low infectious loads suffer severe symptomatic disease [4]. Little is known about the immunology and pathology of this disease in koalas, and it is not understood what factors influence the differing severity of infection in koalas.

Natural Killer (NK) cells are a key part of the mammalian innate and adaptive immune response. These cells can directly lyse, or trigger an immune response to, infected, tumour or abnormal cells. Recognition of these abnormal cells is mediated through NK receptors on the surface of the NK cell. Activation of NK cells is triggered through the balance of activating and inhibitory receptor stimulation [5]. In mammals, these receptors are encoded by two regions of the genome; the leukocyte receptor complex (LRC) which encodes receptors from the immunoglobulin (Ig) superfamily, and the natural killer complex (NKC) which encodes receptors with C-type lectin (CLEC) domains. In eutherians the LRC contains several families including the killer cell immunoglobulin-like receptors (KIRs), leukocyte Ig-like receptors (LILRs) and glycoprotein VI (GPVI). The repertoire of genes within these two families varies greatly between the different mammalian lineages, due to lineage specific expansions and contractions and convergent evolution between the two families [6]. Different eutherian lineages vary in use of these receptors; while primates predominantly use receptors of the LRC, rodents tend to use receptors of the NKC for the same function [6]. However, the most extreme expansions in these families are seen in non-eutherian mammals. Within the monotreme lineage, over two hundred C-type lectin homologs are present, while no LRC receptors have been identified [7]. Conversely, within the marsupials, the opossum has a large expansion of genes within the LRC, but relatively few conserved genes within the NKC [8]. Likewise the Tasmanian devil (*Sarcophilus harrisii*) genome has only a few NKC genes, but many genes within the LRC, although fewer than the grey short-tailed opossum (*Monodelphis domestica*) [9].

Within the NKC are both receptors expressed on NK cells and receptors expressed on other forms of immune cells. NKG2D, encoded by the *KLRK1* gene, is expressed on both T cells and NK cells and plays an important role in tumour response [10]. CLEC4E is a receptor expressed primarily on macrophages [11] and acts as an activating receptor for cell wall components of bacterial species [12]. CD69 is expressed on activated T cells and platelets [13] and may play a role in regulating T cells [14]. CLEC1A is expressed in dendritic cells and regulates T cell activity [15] while CLEC1B is expressed in platelets, monocytes and NK cells [16].

In humans and mice models, it has been demonstrated that NK cells are involved in the response to chlamydial infection [17, 18]. Transcriptome analysis has shown that genes involved in NK activation and cytotoxicity are enriched at the site of infection in children with chlamydial ocular infections compared to healthy controls [19]. In addition to NK receptors, this included perforin (*PRF1*) which is produced by NK cells to lyse infected cells, and natural cytotoxicity receptor 3 (*NCR3*), which increases the efficiency of NK-mediated lysis [20]. Furthermore genes associated with macrophages and T cells were discovered to be upregulated in this study [19].

Whether NK cells or NK cluster encoded receptor genes play a role in the immune response to *C. pecorum* infections in koalas, and the variation in disease severity in koalas, is unknown. Due to the rapid evolution of lineage specific changes seen in the genes encoded in the NKC and LRC, primers and reagents used to study NK receptors in other species are unlikely to have utility in studying koala NK receptors. Therefore we aimed to characterise NK receptors in the koala. We then used this data to design qPCR assays in order to examine the expression of several receptors and genes involved in immune response to chlamydia in a cross-section of *C. pecorum* infected koalas.

## Methods

### Identification of NK receptors

Available koala transcriptomes [21–23] were searched for C-type lectin and Ig superfamily NK receptors, and orthologues to *PRF1* and *NCR3*. TBLASTN [24] searches were conducted using relevant devil, opossum and human sequences. Furthermore, HMMer searches were conducted. To do this, profile hidden Markov models (HMM) were constructed using relevant family members in devil, opossum and human. The 6-frame translation of koala transcriptomes were then searched using the constructed profile HMM using HMMER v3.0 [25]. To confirm their identity, all putative koala NK sequences were used as queries in BLASTP against SWISSPROT. All identified sequences have been deposited into a publicly available database (http://hp580.angis.org.au/tagbase/gutentag/). For phylogenetic tree construction, protein sequences were aligned using MUSCLE [26] through the software package MEGA6 [27], using default parameters. Phylogenetic trees were constructed using the neighbour-joining algorithm, with pairwise deletion of gaps under the p-distance model and evaluation through 1000 bootstrap resamplings in MEGA6.

### Ethics statement

Queensland University of Technology Animal Ethics Committee (Approval No. 0700000845) approved the collection of koala blood samples from wild koalas analysed in this study. Blood samples were collected from wild-caught koalas captured as part of a larger field study in the Moreton Bay region of South-East Queensland, Australia. Samples were collected from anaesthetised koalas by field veterinarians as apart of thorough health evaluations. Sample details are shown in Table 1.

**Table 1:** Infection status, age and gender of sample used in this study

	ID	Age	Gender	qPCR	Western blot
Group 1 (*n* = 11)	1			positive	positive
	2	4	Female	positive	positive
	3	7	Female	positive	positive
	4	9	Female	positive	positive
	5	5	Female	positive	positive
	6	4	Male	positive	positive
	7		Female	positive	positive
	8	3	Female	positive	positive
	9	4	Female	positive	positive
	10	12	Female	positive	positive
	11	5	Female	positive	positive
Group 2 (*n* = 8)	12	3	Male	negative	negative
	13	7	Female	negative	negative
	14	1	Male	negative	negative
	15	4	Female	negative	negative
	16	1	Female	negative	negative
	17	3	Female	negative	negative
	18	3	Male	negative	negative
	19	2	Female	negative	negative
Group3 (*n* = 8)	20	5	Female	negative	positive
	21	3	Female	negative	positive
	22	8	Female	negative	positive
	23	3	Male	negative	positive
	24			negative	positive
	25	2	Male	negative	positive
	26	3	Male	negative	positive
	27	1	Female	negative	positive

### Samples

Samples from 18 koalas analysed in this study were prepared and analysed previously [28]. In the present study, samples from an additional nine koalas were also included from the same koala population that was sampled in this previous study. Briefly, peripheral blood mononuclear cells (PBMCs) were harvested from koala blood samples for use in this study. 5–6 ml of blood was collected in 6 ml EDTA blood tubes from koalas and stored at 4 °C until further processing on the same day. RNA extraction was performed from the PBMCs suspended in Trizol reagent using the RNeasy Mini Kit (Qiagen, Victoria, Australia) according to the manufacturer’s instructions. Swabs were collected from the conjunctiva of the left eye, right eye, urogenital sinus (females) and urethra (males) using aluminium shafted cotton tipped swabs (Copan, Interpath Services, Melbourne). Determination of current and previous *C. pecorum* infection of koalas was performed as described previously [23]. Briefly, to confirm active infection, a qPCR assay targeting the *C. pecorum* 16S rRNA gene was used. Western blots were performed to detect the presence of *C. pecorum* –specific IgG in the koala plasma samples to determine previous exposure to *C. pecorum*. Based on these results, samples were classified into three groups (Table 1) including, those with active infection, determined through positive *Chlamydia* PCR (Group I), those that showed no evidence of present or past *Chlamydia* infection with negative *Chlamydia*-PCR and Western-blots (Group 2), and those with negative *Chlamydia* PCR but positive Western-blot results (Group 3). RNA quality and quantity was assessed on the Agilent 2100 Bioanalyser. Only RNA with RIN >7.0 were used. cDNA was synthesised using the QuantiTect Reverse Transcription Kit (Qiagen).

### NKC receptor qPCR assays

Primers were designed to five reference genes (*GUSB*, *HPRT1*, *YHMAZ*, *TBP*, *ACTB*) and four genes of interest (*CLEC1B, CLEC4E, PRF1, NCR3*; Table 2). Primers were designed using Oligo6. qPCR reactions were performed using 1x Quantifast Sybr Green PCR master mix (QIAGEN), 0.5 μM forward and reverse primer (except for *NCR3* and *PRF1* where 0.25 μM primers were used to prevent primer-dimer) and 50 ng cDNA in a total volume of 20 μl. qPCR reactions were performed in the RotorGene 6000. Reverse-transcription and cDNA negative controls were included in duplicate or triplicate in every run to detect gDNA or cDNA contamination. The qPCR cycle conditions were as follows: an initial denaturation step at 95 °C for 5 min followed by 40 cycles of denaturation at 95 °C for 10 s followed by a combined annealing/extension step at 60 °C for 30 s. Fluorescence signal was acquired at the end of each annealing/extension step. This was followed by a melt cycle to evaluate specific amplification of the product where samples were heated from 50 to 99 °C with fluorescence signal acquired every 1 °C. In order to test reference genes, a subset of samples in the different experimental classes were amplified with each of the six reference genes (the five designed in this study plus *GAPDH* previously designed [22]). NormFinder was used to rank the reference genes based on their expression stability across samples of the treatment groups [29]. The highest two were selected for the following assays. To determine efficiency, standard curves were generated using serial 1:5 dilutions with five dilutions from the dilution series. These dilutions were generated from a sample containing equal parts of cDNA originating from the three sample groups. All samples in the standard curves and experimental runs were run in triplicate and the Cq values fell within the linear quantifiable range for the relevant standard curve. The Pfaffl method of analysis was used to analyse fold changes in the different treatment groups [30]. Data was log transformed to normalise the data and ANOVA was used to determine statistical significance of these changes.

**Table 2:** qPCR primers developed in this study

Gene	Forward primer 5′-3′	Reverse primer 5′-3′
*GUSB*	TGC AGC TCT GTG ACC GAT AC	ACC AAC TCC TCC ATC ACT TC
*HPRT1*	CGT TGG ATA TGC CCT TGA C	GGT CCA GAG GTG AAG TTG ATG
*YHMAZ*	GTC TGG CCT TGA ACT TCT CTG	GCT TCA TCT CCT TGG GTG TC
*TBP*	GGG AAG ATG GTA TGC ACA G	TCA GTA CCA GCC CTT CTA ATC
*ACTB*	GTA CCC AGG CAT TGC TGA C	GCT GGA AGG TGG AAA GAG AC
*PRF1*	CAG GAC GAG TAC AGT TTC AC	GAT GAA CCG ATG GTA GTC
*NCR3*	GGT GAT TGC GAC TTG TCT CTT G	GGG AGC GGA ACT GGA GAA G
*CLEC4E*	TCT ACA TTG GGC TGA CAG AC	GCT CCC CTA TAT CCC AGA AG
*CLEC1B*	TCC TCA CTG TGT TGG TCT TC	GTC CCG TAG AAA CTC TGA TG

## Results

### Koala NKC receptors

**Fig. 1:**
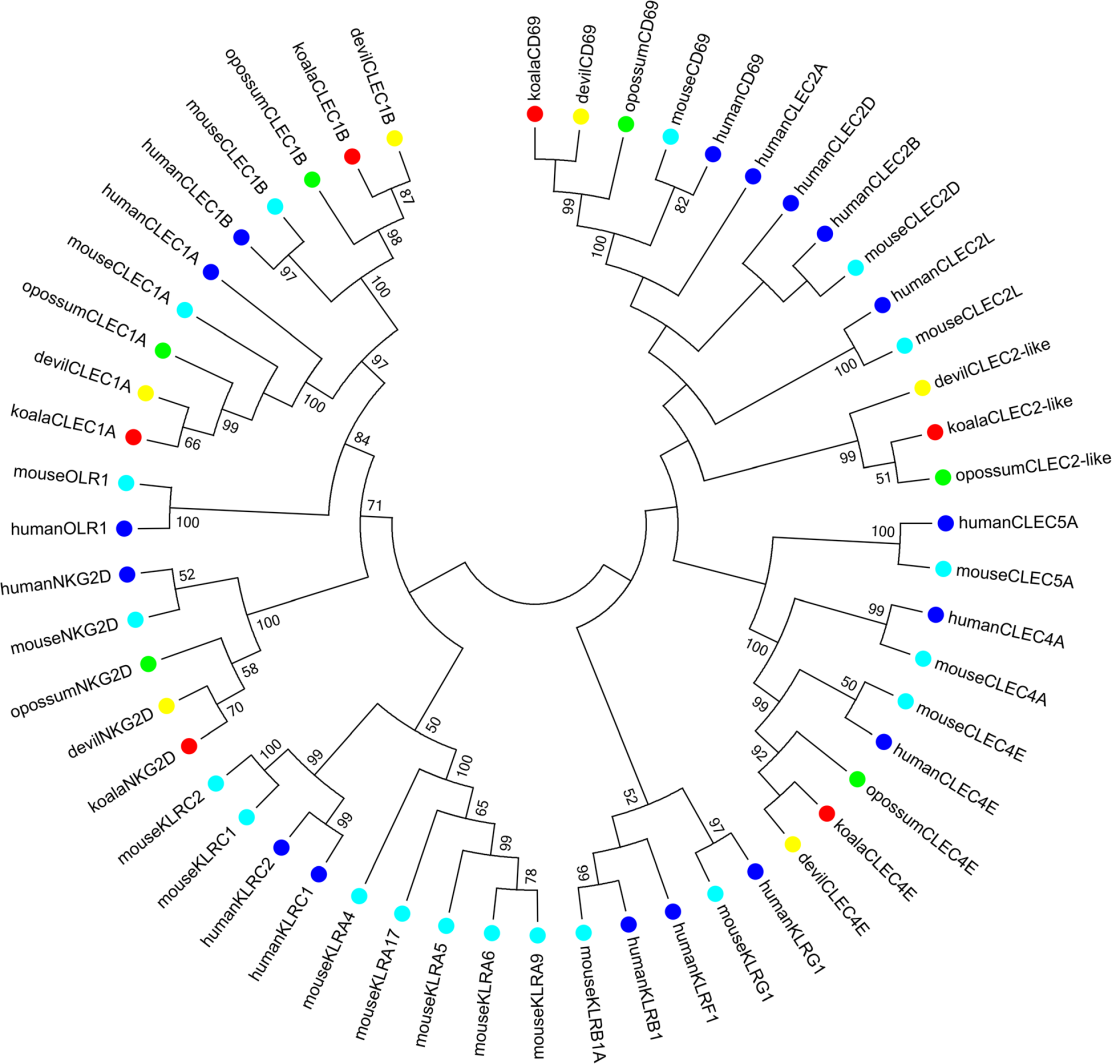
Phylogeny of NKC amino acid sequences. Phylogeny of human (blue), mouse (cyan), opossum (green), devil (yellow) and koala (red) NKC amino acid sequences. Bootstrapped neighbour joining tree, with 1000 bootstrap resamplings. Bootstrap value greater than 50 % only are displayed. Human accession numbers [GenBank: NP_001772, NP_031386, NP_057595, NP_057593, NP_055173, NP_001124183, NP_005118, NP_037401, NP_005801, NP_002249, NP_998823, NP_002251, NP_057607, NP_002534, NP_057268]. Mouse accession numbers [Genbank: NP_001028294, NP_780735, NP_064369, NP_149069, NP_064332, NP_058666, NP_619589, NP_001129540, NP_034783, NP_573466, NP_034781, NP_034867, NP_037401, NP_001163804]. Opossum sequences obtained from IDMM (http://hp580.angis.org.au/tagbase/gutentag/). Devil sequences obtained from [9]

The koala transcriptomes were searched for homologs to mammalian NKC C-type lectin receptors. In both opossum and devil, six C-type lectin NKC receptors have previously been identified [8, 9]. Full length orthologues of each of these genes were identified in the koala transcriptomes (Fig. 1). These genes are direct orthologues to their marsupial and eutherian equivalents, with bootstrap support of at least 99 % for these clades in the phylogeny, with the exception of the CLEC2-like genes. These genes form a single clade in the marsupials, but do not show one-to-one orthology to any mouse or human gene, though do group together with the human and mouse CLEC2 family of genes. No additional C-type lectin NK receptors were identified in the koala transcriptome through BLAST and HMMer searches. As with the other marsupials, fewer genes were seen in this group than in the eutherians, such as humans and mice, or in the monotremes.

### Koala LRC receptors

**Fig. 2:**
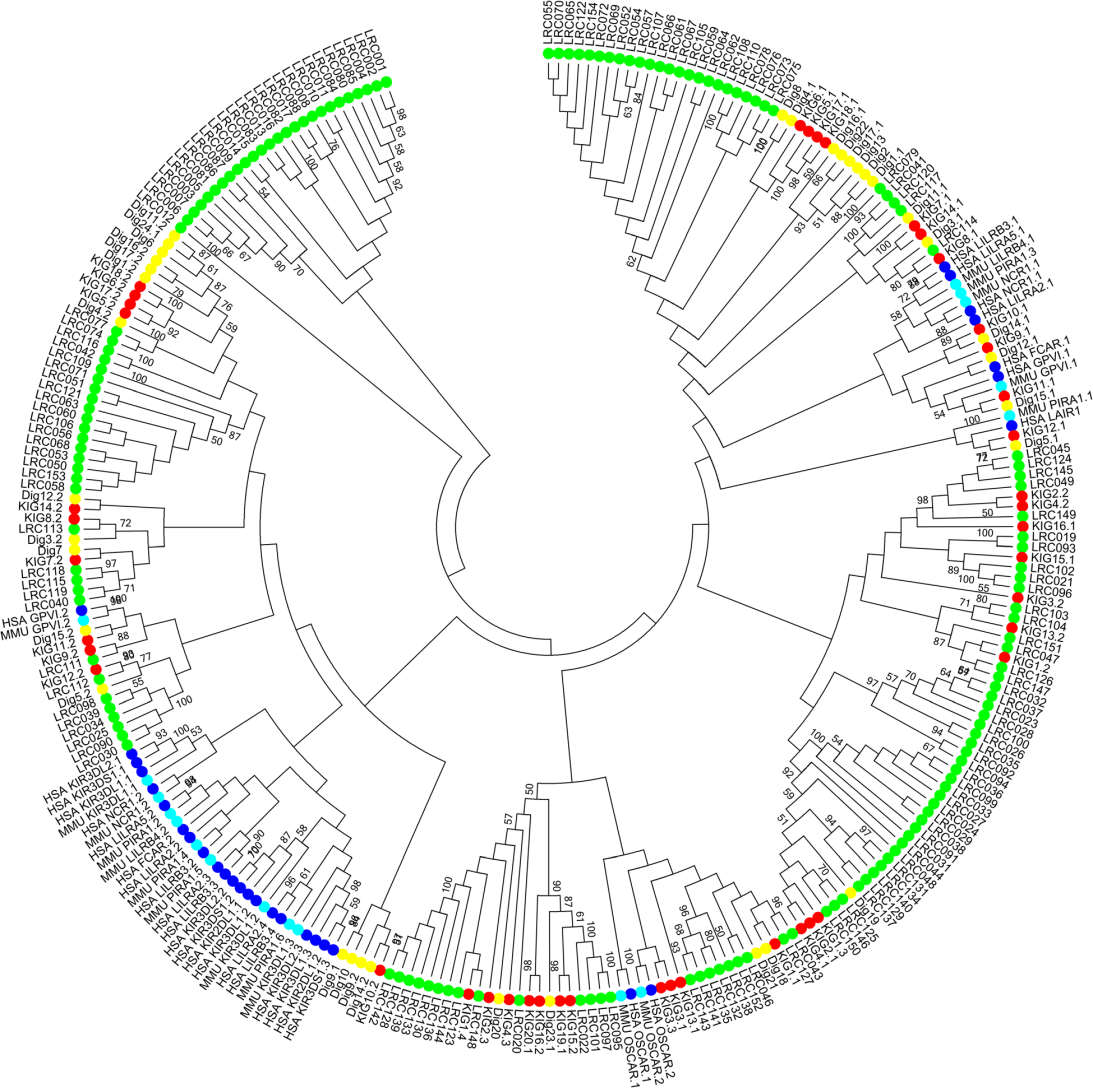
Phylogeny of LRC-Ig like domains. Phylogeny of human (blue), mouse (cyan), opossum (green), devil (yellow) and koala (red) amino acid sequences of LRC Ig-like domains. Bootstrapped neighbour joining tree, with 1000 bootstrap resamplings. Bootstrap value greater than 50 % only are displayed. Human accession numbers [GenBank: NP_004820, NP_001991, NP_001077368, ABU62835, NP_037421, NP_002278, NP_001074919, NP_001124389, NP_006856, NP_067073, NP_006854, NP_006660, NP_005865, NP_006838, NP_036446, NP_036445, NP_055033, NP_056952, NP_036444, NP_996554]. Mouse accession numbers [GenBank: NP_001156486, NP_038560, NP_035217, NP_034876, NP_783440, NP_808417]. Opossum sequences obtained from IDMM (http://hp580.angis.org.au/tagbase/gutentag/). Devil sequences obtained from [9]

**Fig. 3:**
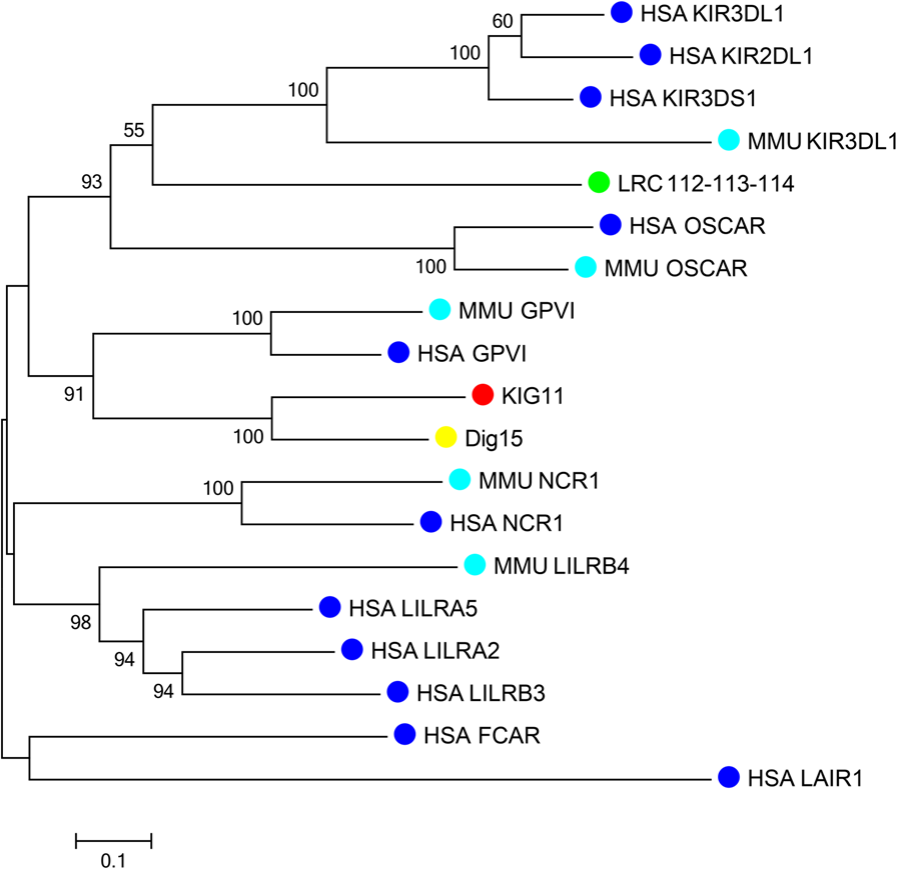
Reduced phylogeny of MII LRC receptors. Phylogeny of human (blue) and mouse (cyan) amino acid sequences of full length MII LRC receptors along with putative GPVI orthologues in opossum (green), devil (yellow) and koala (red). Bootstrapped neighbour joining tree, with 1000 bootstrap resamplings. Bootstrap value greater than 50 % only are displayed. Human accession numbers [GenBank: NP_037421, NP_001991, NP_001124389, NP_067073, NP_001074919, NP_004820, NP_001077368, NP_996554, NP_055033, NP_037421, ABU62835]. Mouse accession numbers [Genbank: NP_783440, NP_034876, NP_808417, NP_001156486]. Opossum sequences obtained from IDMM (http://hp580.angis.org.au/tagbase/gutentag/). Devil sequences obtained from [9]

Twenty-three transcripts encoding NK receptors of the Ig superfamily were identified in the koala transcriptomes. These have been named *‘k*oala *Ig-*like open reading frame*s’*, or KIGs, following the naming used for the devil LRC receptors [9]. The number is very similar to those identified in the devil genome (24), but less than that recognised in the opossum genome (45). The total number of Ig-like domains identified in the koala transcripts was 43, which was greater than the devil 35, but less than that in the opossum (154). In phylogeny (Fig. 2) the identified koala Ig-like domains are interspersed with the other marsupial Ig-like domains, with several small koala expansions seen. A monophyletic clade is formed by mouse and human KIR and LILR domains 2–4 (known as MII type LRC domains) [24] with no marsupial Ig domains within this clade. Likewise, mouse and human LIR domains are found in a single clade with no marsupial sequences. However, koala and devil Ig domains are interspersed among the clade of eutherian Ig domains including domain 1 from *LIR*, *NCR1*, *FCAR*, *GP6*, *LAIR* and *PAIR* genes. This group of eutherian genes are known as MI type LRC domains [31]. One possible orthologous relationship was seen between koala and eutherian Ig domains in the phylogeny; the first Ig domain of KIG11 and DIG15 formed a single clade with the first domain of human and mouse GPVI, and likewise the second Ig domain of these four sequences also formed a single clade. To examine this relationship further, a phylogeny was constructed with the full length sequences of these four peptides as well as a putative orthologue in opossum and full length peptide sequences of other human and mouse LRC receptors (Fig. 3). This phylogeny provides strong support for an orthologous relationship between the four genes with 91 % bootstrap support. Additionally KIG11 shows reasonable sequence identity to the human and mouse GPVI, at 32.5 and 34.6 % amino acid identity, respectively. The putative opossum GPVI orthologue did not group with the GPVI sequences in the phylogeny. The KIGs identified had between one and five domains within the reading frame, with the majority having two domains (61 %). This is similar to opossum where one to five domains per gene was predicted. However all the devil DIGs contained only one to two domains. This difference may be due to the more fragmented nature of the devil genome compared to the opossum, leading to missing DIG domains. As the majority of koala transcripts identified were full length sequences with both start and stop codons, fewer domains are likely to be missing. Entire genes may be missing from our analysis however if they were not expressed in the transcriptomes studied and therefore genomics will be required to fully catalogue the LRC NK receptors in the koala.

### NCR3 and PRF1 sequences

**Fig. 4:**
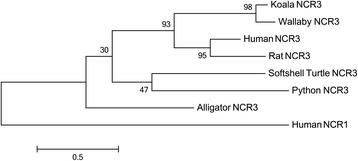
Phylogeny of NCR3 amino acid sequences. Human NCR1 is used as an outgroup. Bootstrapped neighbour joining tree, with 1000 bootstrap resamplings. NCR3 sequences; human [Swiss-Prot: O14931], rat [Swiss-Prot: Q8CFD9], softshell turtle [GenBank: XP_008175878], python [GenBank: XP_007443990], alligator [GenBank: XP_006258277]. Human NCR1 [Swiss-Prot: O76036]

**Fig. 5:**
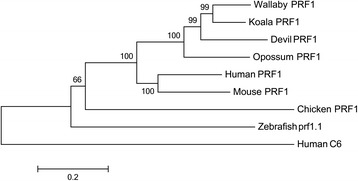
Phylogeny of PRF1 amino acid sequences. Human C6, a family member, is used as an outgroup. Bootstrapped neighbour joining tree, with 1000 bootstrap resamplings. PRF1 sequences; wallaby [Ensembl: ENSMEUT00000016158], devil [Swiss-Prot: G3WB83], opossum [Swiss-Prot: F7FU19], human [Swiss-Prot: P14222], mouse [Swiss-Prot: P10820], chicken [Swiss-Prot: R4QNW4], zebrafish [Swiss-Prot: E7FA66]. Human C6 [Swiss-Prot: P13671]

Full length transcripts of *NCR3* and *PRF1* were identified in the koala transcriptomes. Interestingly, *NCR3* is a pseudogene, or missing from many species. It has found to be a pseudogene in the mouse [32] and additionally it was not found in searches of the opossum genome [33]. In addition it has not been previously identified in the devil or platypus. In order to determine whether it was present in these genomes, we performed BLAST searches of these genomes using the koala and human *NCR3* sequences, but no orthologue was discovered in these two genomes. We did however identify *NCR3* in the Tammar wallaby genome (Fig. 4). A phylogeny of koala NCR3 is shown in Fig. 4, with strong bootstrap support (78 %) grouping the koala and wallaby sequences with human and rat sequences. As well as being missing from the devil, opossum and platypus, NCR3 has not been identified in any bird, amphibian or fish genome. However, several orthologues have been identified in reptile species including alligator, turtle and python, by automatic annotation of reptile genomes in Ensembl and NCBI, demonstrating that this gene did not originate in mammals but has an ancient origin dating back to the Mesozoic Era. Unlike *NCR3*, *PRF1* is present in most vertebrate genomes available, a selection of which is included in Fig. 5 along with the identified koala sequence. Strong bootstrap support (100 %) indicates that this is an orthologue of the human and mouse PRF1 peptides.

### Development of qPCR assays

Primer sets were developed for five reference genes (*GUSB*, *HPRT1*, *YHMAZ*, *TBP*, *ACTB*; Table 2). The five reference genes were tested, along with the previously developed *GAPDH* primer set. In order to select reference genes for the assay, NormFinder was used to rank the reference genes based on their expression stability across samples of the treatment groups. The two highest ranked reference genes, *YHMAZ* and *HPRT1*, were selected for use in the qPCR assays. Initially primers were designed for 16 genes of interest which include genes of the LRC, NKC and genes related to the NK cell pathway, with a focus on genes previously demonstrated to be upregulated in chlamydia [19, 34]. Standard curves were produced for all 16 and four were selected for qPCR trials based on sufficient expression and primer quality (*CLEC1B, CLEC4E, PRF1, NCR3*; Table 2). All primer sets were designed so that the amplicon spanned an exon boundary to prevent amplification of contaminating gDNA. All genes had standard curves with an R^2^ of > 0.98 and had greater than 90 % efficiency. This provides assays that can be used for quantification of four genes in koalas, as well as five new reference genes which can be used in further koala qPCR assays.

### Expression of *CLEC1B*, *CLEC4E*, *NCR3*, and *PRF1*

**Fig. 6:**
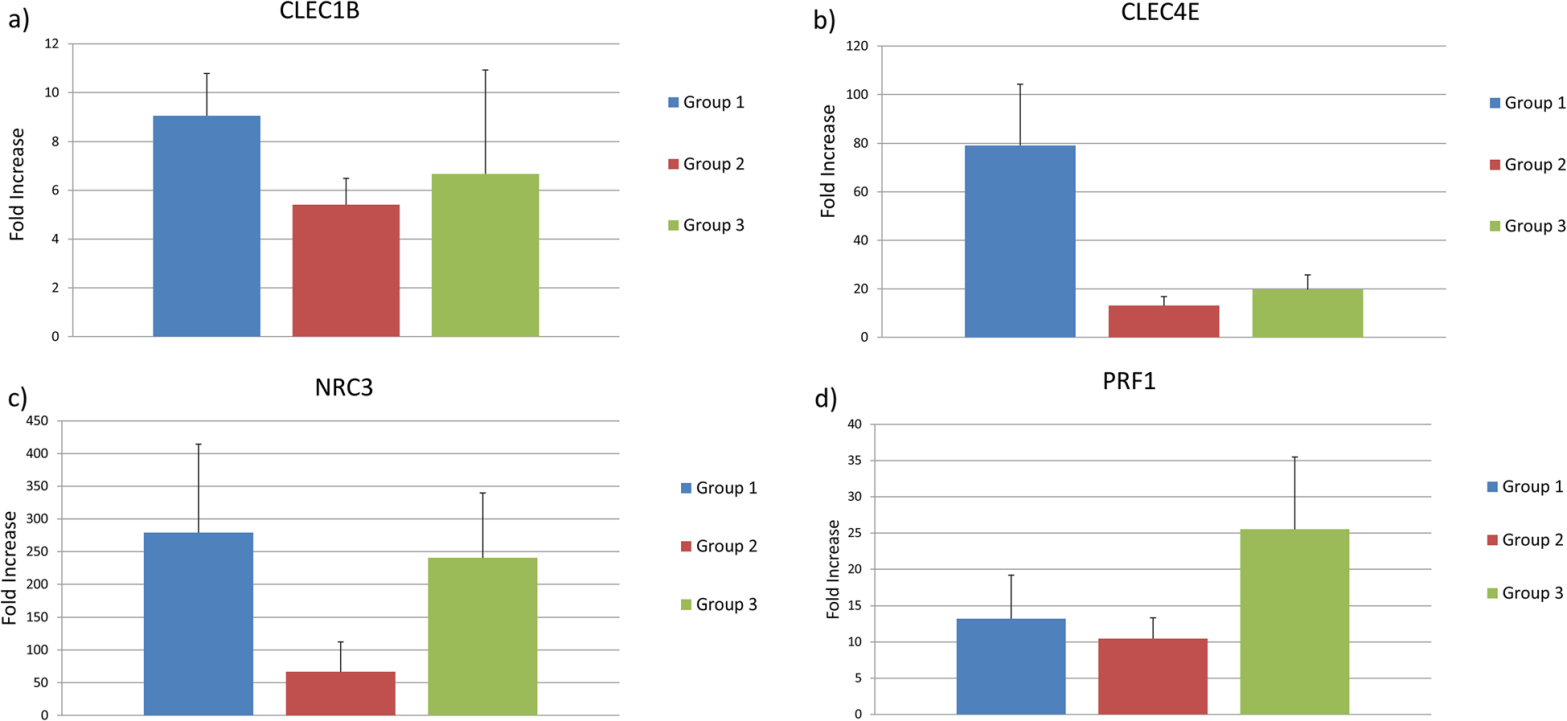
Relative expression of four NK pathway genes in koalas. Relative expression of CLEC1B (**a**), CLEC4E (**b**), NCR3 (**c**) and PRF1 (**d**) in Group 1 (Chlamydia-PCR positive), Group 2 (Chlamydia-PCR and Western blot negative koalas) and Group 3 (Chlamydia PCR negative but Western blot positive koalas))

The expression of two CLEC-like receptors and two genes involved in NK cell lysis was examined in koalas with current evidence of chlamydial infection (Group 1), koalas with past infection based on the presence of *Chlamydia*-specific IgG but were negative to the *Chlamydia* PCR (Group 3) and koalas that were negative to both *Chlamydia*-PCR and western blot analysis of koala plasma (Group 2). Of the four genes assayed, one gene, *CLEC4E* had a significant *p* value (*p* = 0.032) in the ANOVA test, with the mean relative expression of Group I (40.6; SD 3.8) being significantly higher than the mean expression of the Group 2 (9.6; SD 3.1; Table 3; Fig. 6). This is roughly a four-fold increase in expression of this gene in Group I and indicates that cells with this receptor are at a higher concentration in the blood of Group I koalas. Significant upregulation was not seen in any of the other genes, although it must be noted that low sample numbers, and high variance may have resulted in a smaller effect being undetected. The remaining three genes did show a trend for higher mean expression in Group I than Group 2 koalas. The trend was strongest in *NCR3* and *CLEC1B* where there was an approximately five fold and fourfold increase in expression in Group I koalas, respectively. In addition, a significant difference between Group 3 koalas and either Group I or Group 2 was not seen in any of the genes.

**Table 3:** Mean, standard deviation and *p* value of qPCR expression study

Gene	Mean			Standard Deviation			*p* value
	Group 1	Group 2	Group 3	Group 1	Group 2	Group 3	
*CLEC1B*	7.6	4.74	5.91	1.86	1.82	1.63	0.229
*CLEC4E*	40.6	9.57	17.45	3.88	3.09	2.04	0.032
*NCR3*	24.95	4.45	87.47	19.78	1.98	8.27	0.061
*PRF1*	4.35	3.91	14.05	5.22	2.27	3.45	0.122

Mean expression, standard deviation and *p* value of *CLEC1A*, *CLEC4E, NCR3* and *PRF1*, in Group 1 (koalas with active infection), Group 2 (koalas with no infection and no antibodies) and Group 3 (koalas with antibodies but no active infection)

## Discussion

Despite koalas being threatened by a range of infectious diseases, little is known about the immune system of koalas. In recent years, studies have begun to investigate koala immunogenetics, with MHC [35–37], TLR [38] and a range of cytokines [22, 23, 39] having been the focus of investigations. We have now added to this by investigating the receptors of the NKC, LRC and NK cell associated genes of the koala, developing qPCR assays to characterise expression of four key genes, and providing preliminary analyses into the regulation of these in *C. pecorum* infected animals. This study has shown that transcriptomes represent an alternative to genomics for identification of genes, including those of rapidly evolving and divergent families. All expected members of the NKC and a similar number of Ig-like domains to the devil were identified from the koala transcriptomes.

From previous studies in the opossum and koala, we expected to find orthologues to six therian NKC genes, *CLEC1A, CLEC1B, CLEC4E, KLRK1, CD69,* and *CLEC2-like*, and we were successful in identifying full length transcripts for each of these genes in the koala. No additional genes within this family were identified, including *OLR1* which is conserved across monotremes and eutherians, but has not been identified in marsupials [8, 9]. All three of the marsupial species investigated have this minimal conserved NKC and this may represent the norm for the marsupial NKC. This is in contrast to the eutherians, where the number and composition of genes within this group can vary greatly, as well as to the monotremes which have a huge expansion of genes in this family.

Previous studies in marsupials have shown an expansion in LRC genes which generally lack orthology to eutherian LRC receptor genes [8, 9]. Twenty-three KIGs were identified in the present study, similar to the number of DIGs found in devil (23) though less than the number found in the opossum (45). It is possible that additional genes are present in the koala genome that weren’t expressed in the koala transcriptomes. However, one advantage of the transcriptomic approach is that un-expressed pseudogenes would not be identified, so our gene set may be a more accurate reflection of the number of functional LRC genes than those identified from the devil and opossum genomes. Expressed but non-functional pseudogenes may also be included in our predicted koala LRC set, and so functional studies will be required to conclusively determine which genes in koalas are functional. An additional advantage of use of transcriptome data over genomic data is due to the difficulty in assembly and annotation of multigene families in genomes. This is especially the case with LRC receptors where genes can be made of varying number of Ig domains, which make determining full gene sequences from genomic data highly challenging. For example many single LRC Ig domains were identified in the devil on short scaffolds which likely form only part of full LRC genes [9]. With transcriptomic data we were able to identify full length LRC genes.

The expansion of LRC genes is consistent across the marsupials investigated and this suggests that marsupials may rely primarily on Ig-like NK receptors rather than C-lectin NK receptors. The LRC domains found in the koala, like in the devil and opossum, generally lack orthology to eutherian LRC domains. This suggests that this family is rapidly evolving in these different lineages. Only one orthologous relationship was identified in this study; a koala sequence showed orthology to eutherian GPVI. GPVI in eutherians is present on the surface of platelets and acts a signalling receptor for collagen [40]. This protein may have a conserved role among mammals explaining its presence across marsupials and eutherians. Previously an opossum sequence was suggested to be orthologous with GPVI [8]. However this sequence did not group with GPVI either in the single Ig domain phylogeny or in the full length sequence phylogeny. However, only a partial prediction exists for this opossum sequence and a full length sequence may allow for the identity of this sequence to be fully resolved. Some domains showed possible orthology across the three marsupial species, while other domains clustered into opossum, devil or koala specific expansions. This suggests that some LRC domains may date back to the last common ancestor of extant marsupials while others have duplicated within the Australian or South American marsupial lineage.

In addition to these families, two genes of the NK pathway, which have previously been linked to response to chlamydial infection in humans, were identified in the koala and compared to orthologous sequences in other vertebrates. *NCR3* appears to have a complex history, with an origin dating at least to the last common ancestor of mammals and reptiles, but the gene appears to have been lost in many lineages, with only a pseudogene present in mice, and the gene not being identified in opossum, devil, platypus or any bird genome. *PRF1* is more conserved being present in all the genomes examined in this study.

In preliminary investigation of expression of four genes of interest, significant upregulation of one gene (*CLEC4E*) was seen in koalas with *C. pecorum* infection, compared to those without infection based on Chlamydia PCR. CLEC4E is a receptor expressed primarily on macrophages [11]. In eutherians, CLEC4E is known to be an activating receptor for cell wall components of bacterial species and damaged cells [12]. CLEC4E is highly induced by lipopolysaccharide [41], an active pattern recognition receptor ligand in chlamydia [42]. Therefore it is possible that this receptor is able to recognise chlamydial cells or *Chlamydia*-infected cells in animals that are currently shedding *C. pecorum* to induce an immune response. In addition, the remaining three genes showed higher mean expression in the diseased than non-diseased koalas, though these were not statistically significant. The trend was strongest in *NCR3* and *CLEC1B* where there was an approximately five fold and fourfold increase in expression in the diseased koalas, respectively. As with previous studies of gene expression in *Chlamydia*-infected koalas [15, 16] and with other studies of immune gene expression in diseased populations of other wild species [43, 44], a large variability was seen in the expression of these genes in the non-diseased, but particularly in the diseased koalas. This is not surprising as the koalas used in this study come from a cross-sectional cohort of animals with a range of symptoms, stages of disease progression, ages, genders and reproductive statuses. To overcome this variability a larger sample set may be needed. Additionally, future studies should target mucosal samples of the infected sites, in order to characterise the response at the site of infection. As studies in other species have demonstrated early recruitment of NK cells to the site of infection [18], there is likely to be more significant difference in the density of NK cells and associated genes at the site of Chlamydia infection in koalas. Additionally, it would be of interest to investigate differences in expression of these genes in koalas with differing responses to infection and disease outcomes. We plan to tackle these questions using transcriptomics approaches rather than single gene qPCRs. This will be possible now that we have a good annotation of complex immune gene families, including the NK receptor clusters.

## Conclusions

In this study genes of the NKC, LRC and genes of the NK pathway were identified and characterised in the koala. The repertoire of NK genes in devil was similar to the devil, though species specific differences including gene expansions and deletions were identified. Four genes of interest were examined using qPCR assays in koalas with chlamydia. We found evidence that one receptor (CLEC4E) may be upregulated in koalas with chlamydia and found a non-significant trend for upregulation of several NK genes. By continuing to build a picture of the immunology of the host response to chlamydial infection and by exploring whether immune response may underlie the differing symptomatic responses seen in koalas to chlamydia, we may be able to predict the outcome of infection. Such prognostic tools will be valuable in assessing the impact of these infections on different Chlamydia infected koala populations, information that can be used to make informed decisions about the management of this dwindling iconic native species.

### Availability of supporting data

The data sets supporting the results of the article are available in the Immunome Database for Monotremes and Marsupials [http://hp580.angis.org.au/tagbase/gutentag/].

## Abbreviations

NK: Natural killer
LRC: Leukocyte receptor complex
NKC: Natural killer complex
Ig: Immunoglobulin
CLEC: C-type lectin
KIRs: Killer cell immunoglobulin-like receptors
LILRs: Leukocyte Ig-like receptors
GP: Glycoprotein
IFNγ: Interferon gamma
PRF: Perforin
NCR3: Natural cytotoxicity receptor 3
HMM: Hidden Markov models
MHC: Major histocompatibility complex
TLR: Toll-like receptor
